# Idiopathic Effusive Constrictive Pericarditis in a Young Patient: Early Diagnosis and Curative Surgical Treatment

**DOI:** 10.7759/cureus.91081

**Published:** 2025-08-27

**Authors:** Alina Mariana Paraschiv, Despina M Toader, Cristina Florescu, Diana Rodica Tudorascu, Tudor Adrian Balseanu

**Affiliations:** 1 Cardiology, Filantropy Hospital, Craiova, ROU; 2 Cardiology, Craiova Cardiology Center, EuroEchoLab, Craiova, ROU; 3 Cardiology, Faculty of Medicine, The University of Medicine and Pharmacy, Craiova, ROU; 4 Internal Medicine, Faculty of Medicine, The University of Medicine and Pharmacy, Craiova, ROU; 5 Physiology, Faculty of Medicine, The University of Medicine and Pharmacy, Craiova, ROU

**Keywords:** constrictive pericarditis, echocardiography, idiopathic, pericardiectomy, treatment

## Abstract

We will present the case of a young man with a respiratory condition lasting for several months, treated with anti-inflammatory drugs and antibiotics, who, following an abdominal ultrasound that revealed pericardial fluid, was referred to the Emergency Department of Filantropy Hospital. Serological tests and imaging investigations were performed to establish the diagnosis and etiology. The first imaging impact was the echocardiogram, which revealed suggestive features of constrictive pericarditis, later confirmed through cardiac magnetic resonance imaging. Due to advanced symptoms, right heart failure phenomena, with hepatomegaly and grade 3 Lewis jugular vein distension, the initial treatment was with intravenous loop diuretics at a low dose and a sodium-glucose cotransporter-2 (SGLT2) inhibitor, but with poor tolerance due to a drop in blood pressure. For this reason, the patient was urgently referred to the Cardiovascular Surgery Department, where the case was resolved through pericardiectomy. The histopathological examination did not reveal a specific etiology as the cause of the constrictive pericarditis. The patient's postoperative evolution and paraclinical investigations showed a favorable status. Timely surgical intervention for this patient made the difference between symptom worsening and the potential onset of complications, and the successful resolution of the case.

## Introduction

Effusive-constrictive pericarditis (ECP) is a rare pathology of the pericardium. Among individuals undergoing pericardiocentesis, ECP was identified in roughly 5.8% of cases. The prevalence was slightly higher, around 6.8%, in patients presenting with clinical tamponade. In contrast, among all patients with pericardial disorders, the occurrence of ECP was approximately 0.93% [1]. This was first described in the literature in 1971 by Hancock. Its etiology is very difficult to establish, so the later it is detected, the lower the probability of identifying a clear etiology. A viral or tuberculous pericarditis, undiagnosed in the initial phase, could explain the cases of some patients in this group, in which case it is most often classified as idiopathic [2].

Echocardiography and Cardiac Magnetic Resonance Imaging (MRI) are essential investigations to establish a probable diagnosis of constrictive pericarditis. The criteria for establishing this diagnosis include changes in the pericardium, such as thickening, septal bouncing, changes in the transmitral flow in relation to respiration, as well as dilation of the inferior vena cava [1]. In the following case, we will discuss the etiology, symptoms, paraclinical investigations, as well as the therapy of effusive constrictive pericarditis in a young man.

## Case presentation

We discuss the case of a 41-year-old Caucasian male patient from an urban environment, a smoker for approximately 20 years, who has been abstinent for 14 days, an animal and travel enthusiast, vaccinated against COVID-19 with the Johnson & Johnson vaccine during the COVID-19 pandemic, with numerous respiratory issues in the past four months, treated with multiple courses of antibiotics and non-steroidal anti-inflammatory drugs (NSAIDs) such as ibuprofen, but without serological tests or specialized medical reevaluation. He presents to the Emergency Department of Filantropy after an abdominal ultrasound revealed a large pericardial effusion, complaining of dyspnea on minimal physical exertion, marked physical fatigue, diffuse abdominal pain, unintentional weight loss of approximately 3-4 Kg in the past month, palpitations, and a cough with mucous expectoration, with symptoms progressively worsening in the past week. There is no reported contact with tuberculosis or other infectious-contagious diseases in the family. No known hereditary or genetic disorders reported in the patient's family history. The physical examination reveals a conscious, cooperative patient with altered general condition, normoweight (BMI=23 kg/m²), slightly pale skin, anxious and suffering facial expression, blood pressure 110/70 mmHg, heart rate 100 bpm (regular), SaO2 95-96% without oxygen, rhythmic and tachycardic heart sounds, distended jugular veins. Pulmonary examination revealed decreased breath sounds bilaterally at the bases, scattered crackles and sub-crackles bilaterally at the bases. Liver with the lower margin 3-4 cm below the costal margin, spleen within normal limits. Peripheral pulse filiform, temperature 36.8°C at presentation (fever and chills for the last 3-4 days, treated with levofloxacin). The patient had a condition of diuresis (2100 ml/24h).

**Figure 1:**
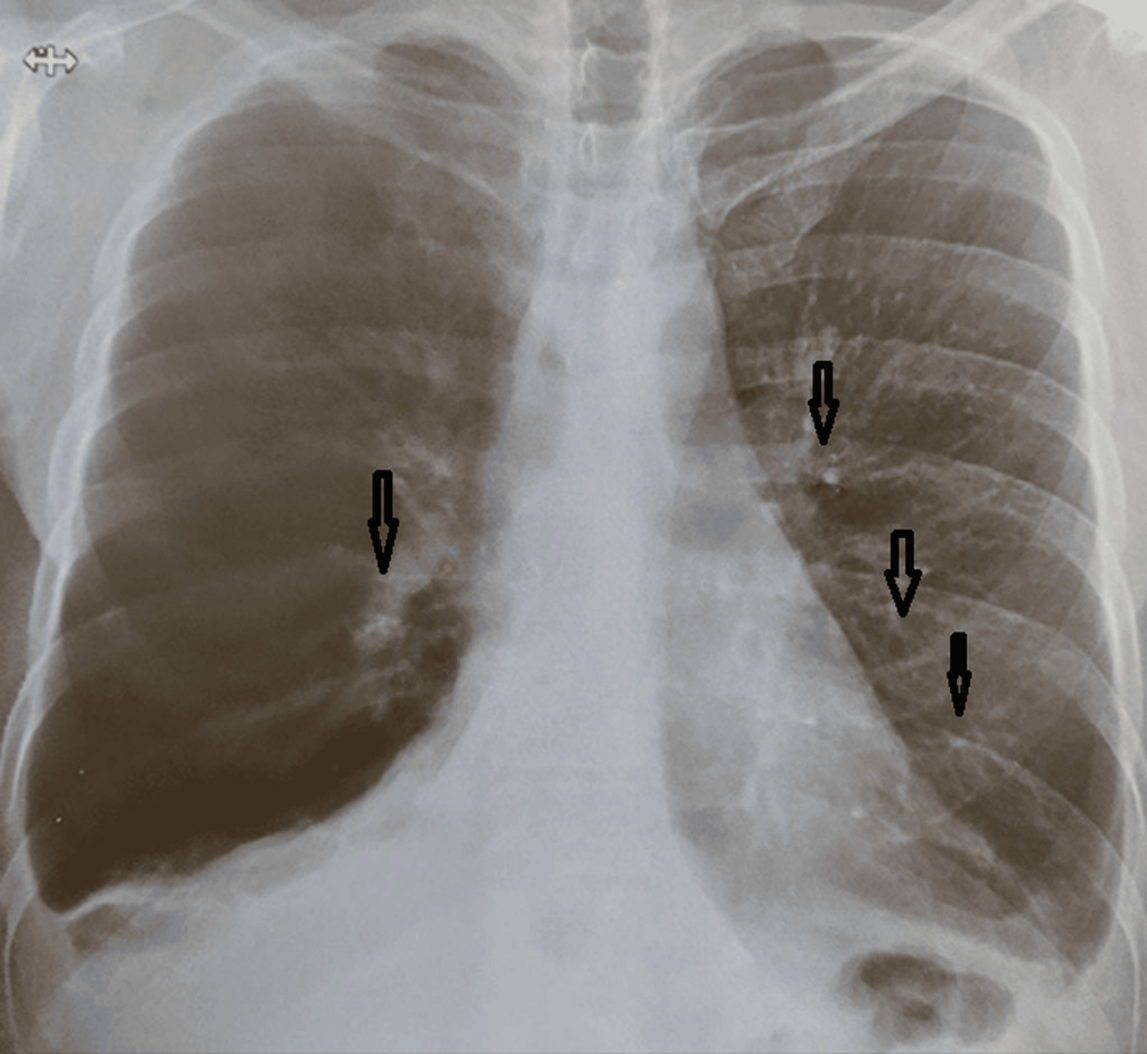
Chest X-ray The chest-pleuro-mediastinal-cardiopulmonary radiograph upon emergency presentation.

The chest-pleuro-mediastinal-cardiopulmonary radiograph, according to Figure 1, revealed a cardiothoracic ratio < 0.5, reticulo-micro-nodular opacities distributed bilaterally at the hilum and bases, more pronounced on the left, with occupation of the left and right costo-diaphragmatic spaces.

Laboratory tests (Table 1), revealed negative markers of myocardial necrosis evaluated dynamically, with no enzymatic shift, significantly elevated inflammatory markers, C-reactive protein, fibrinogen, erythrocyte sedimentation rate (ESR), procalcitonin, normal thyroid function, normal renal function, natriuretic peptides within normal limits, hypoproteinemia, 6 g/dl, mildly decreased hemoglobin, 10,7 g/dl, negative rapid SARS-CoV-2 antigen test, negative influenza A and B test, negative sputum examination, negative rapid Helicobacter pylori antigen test, negative HIV test, no occult bleeding, and negative Quantiferon test. The autoimmune screening did not provide conclusive information to support an autoimmune etiology. Negative tumor markers. Interferon gamma release assay negative, intradermal reaction to tuberculin negative.

**Table 1:** Laboratory test report NT-proBNP: N-terminal pro B-type natriuretic peptide, CK-MB: Creatine Kinase-MB Isoenzyme, ESR: Erythrocyte Sedimentation Rate.

Variable	The reference value	Patient value
NTproBNP	0-125 pg/mL	121 pg/mL
Troponin I	0.01-0.06 ng/mL	0.01 ng/mL
CK-MB	1-24 ng/mL	20 ng/mL
Hemoglobin	14-16 g/dL	10.7 g/dL
C-reactive protein	<0.5 mg/dL	10.41 mg/dL
Fibrinogen	200-400 mg/dL	703 mg/dL
ESR	1-10 mm/h	115 mm/h
Procalcitonin	<0.05 ng/mL	0.173 ng/mL
Total protein	6.6-8 g/dL	6 g/dL
Urea	<43 ng/dL	22 mg/dL
Creatinine	0.74-1.35 mg/dL	0.66 mg/dL

**Figure 2:**
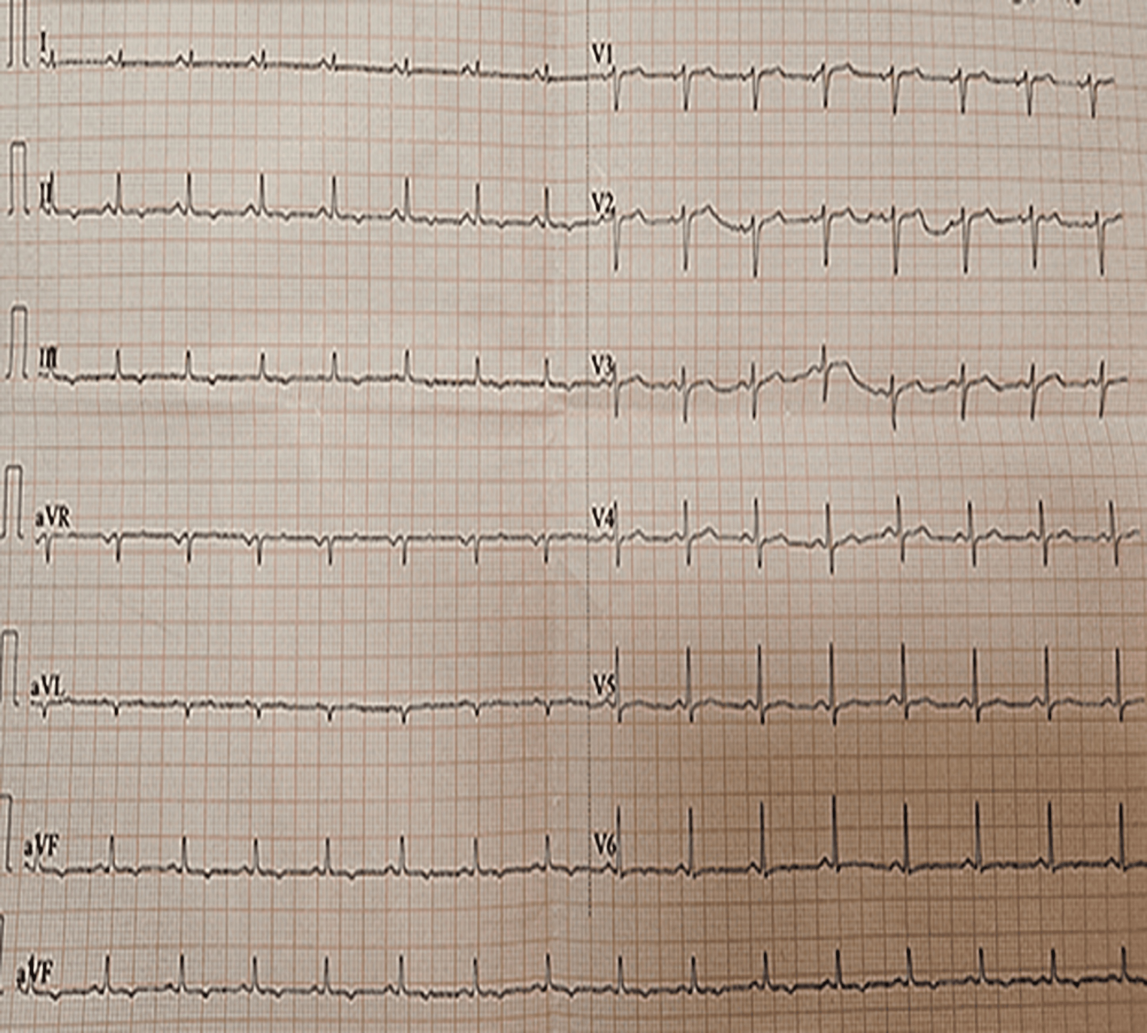
Electrocardiogram The Electrocardiogram (ECG) showed sinus rhythm, heart rate 93 bpm, PR = 120 ms, low-voltage complexes in the limb leads, and a negative T wave in leads II, III, and augmented vector foot (aVF).

The ECG showed sinus rhythm, heart rate 93 bpm, PR = 120 ms, low-voltage complexes in the limb leads, and a negative T wave in leads II, III, and augmented vector foot (aVF) (Figure 2).

Upon conducting a 24-hour Holter ECG, a normal sinus rhythm was observed throughout the monitoring period with no arrhythmias or conduction disturbances. Heart rate ranged between 85 and 123 bpm, with no significant R-R pauses (>2000 ms). Normal heart activity, no arrhythmias, and a stable rhythm were observed. Spirometry showed normal values, with FEV1 at 99% (normal forced expiration), TLC at 118% (slightly increased total lung capacity), and VC at 86% (slightly reduced vital capacity). Overall, a normal lung function was observed, with minor variations within acceptable limits.

Echocardiography and Transesophageal Echocardiography (TEE): Pericardial thickening was detected with moderate pericardial fluid present in the infero-posterior portion. Fibrin strands are visible within the pericardial cavity. The left ventricle is small in size, with altered global systolic function, an estimated Left Ventricular Ejection Fraction (LVEF) of 40% due to diffuse hypokinesia of the left ventricular walls. Paradoxical motion of the interventricular septum is present. Changes corresponding to respiration in the interventricular septum are noted, with protrusion towards the left ventricle during inspiration and towards the right ventricle during expiration. The inferior vena cava is dilated, with spontaneous contrast seen and no inspiratory collapse. The hepatic veins are dilated with respiratory variations.

**Figure 3:**
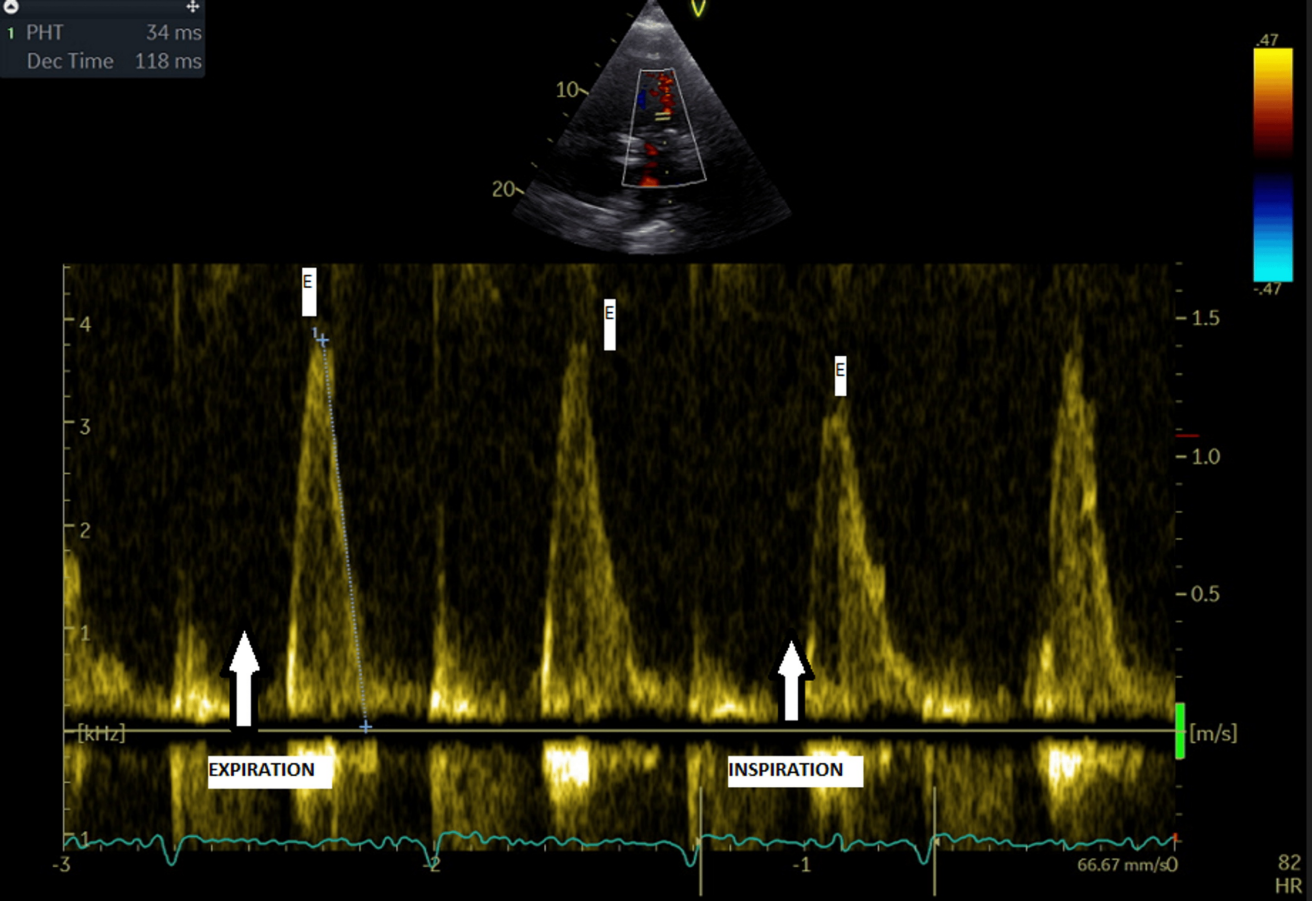
Echocardiography Pulsed Doppler transmitral shows an E wave velocity of approximately 1.5 m/s, with a deceleration time of the E wave of 118 ms, this respiratory variation is a characteristic sign of constrictive pericarditis

The pulsed-wave Doppler of the transmitral flow shows a higher E wave, a smaller A wave, and a restrictive type diastolic dysfunction pattern, with respiratory variations, including a decrease in the E wave during inspiration compared to the initial value (Figure 3).

**Figure 4:**
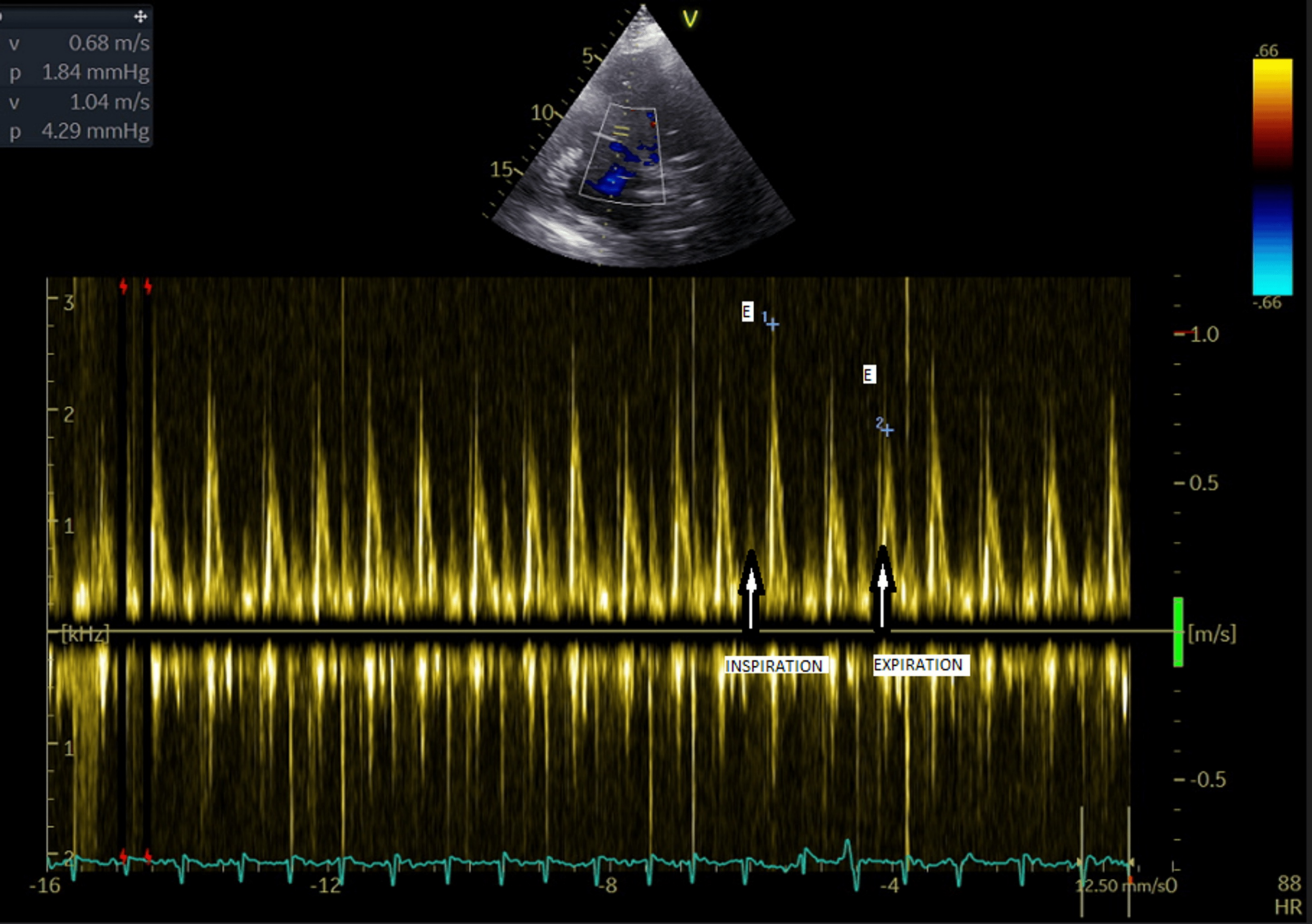
Echocardiography Pulsed Doppler at the level of the tricuspid valve with respiratory variations. This pattern of respiratory variation is frequently associated with constrictive pericarditis.

The transtricuspid flow shows a higher E wave than the A wave, with reversed changes compared to the transmitral flow, with an increase in the E wave during inspiration compared to the baseline value (Figure 4). 

**Figure 5:**
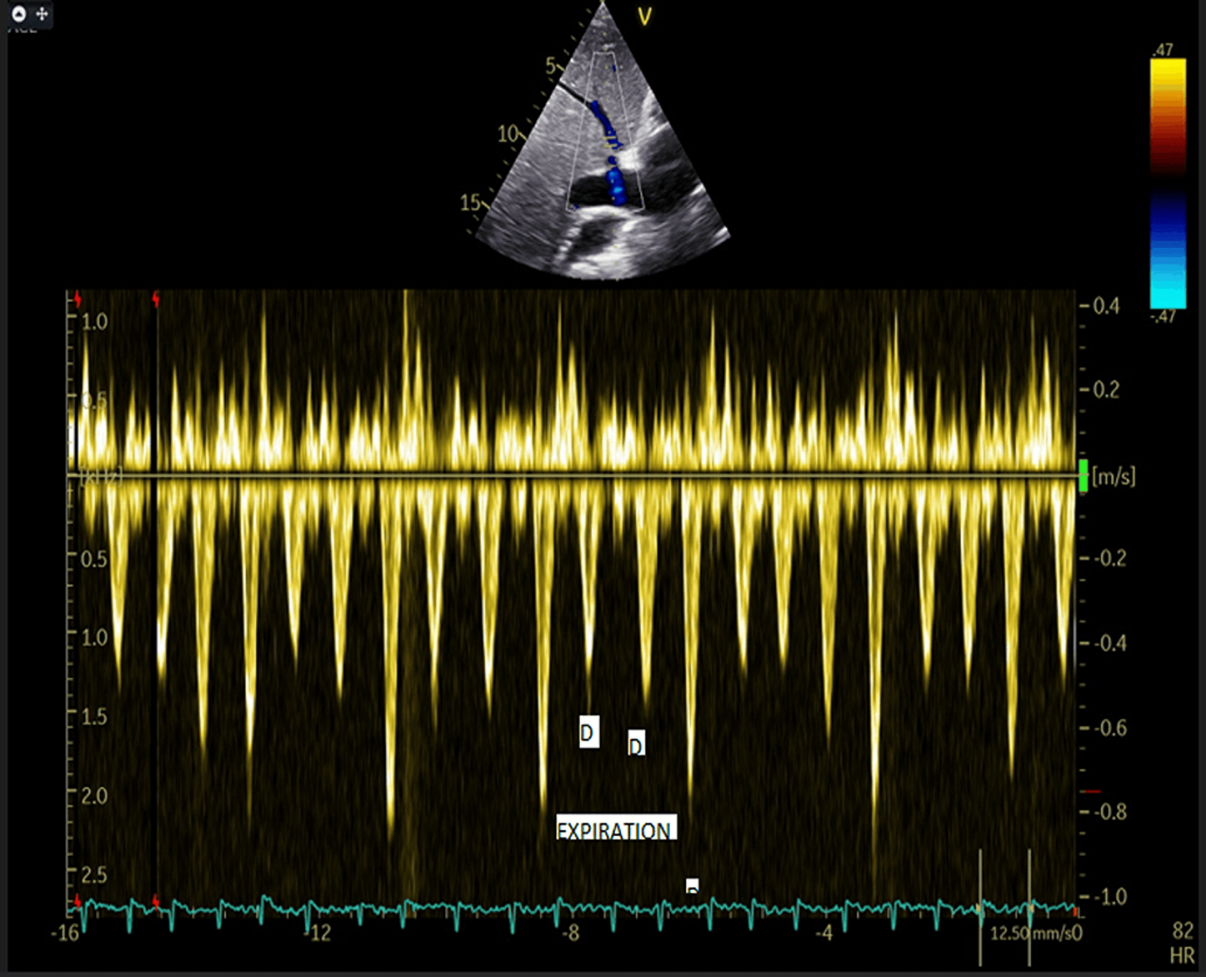
Echocardiography The subcostal view at the level of the hepatic veins shows respiratory variations

The flow in the hepatic veins reveals a slightly increased velocity during inspiration for the S and D waves, and a decrease in the velocity of the D wave during expiration (Figure 5).

**Figure 6:**
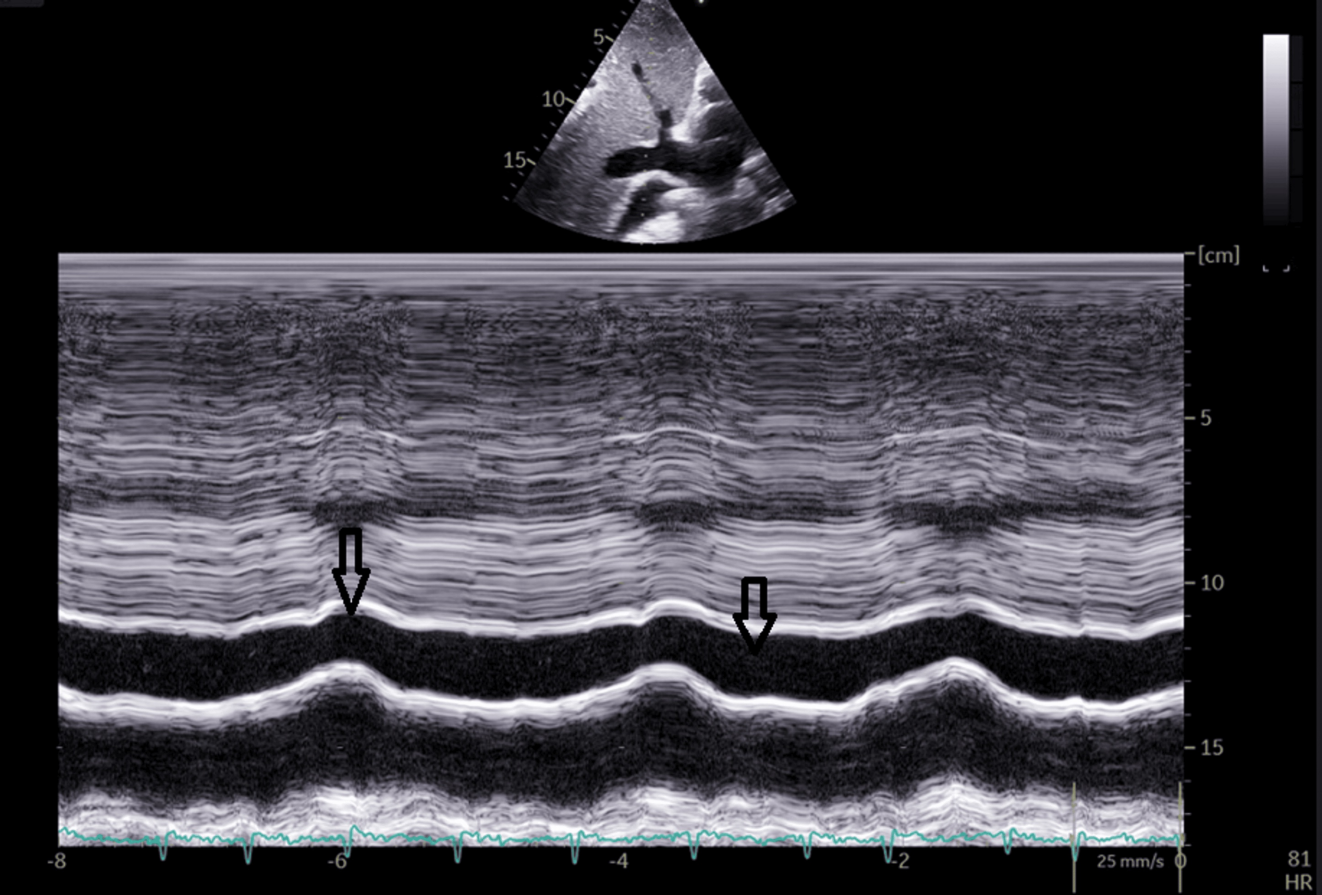
Echocardiography In the subcostal view, in M-mode, the enlarged inferior vena cava without inspiratory collapse is highlighted

On tissue Doppler examination, the velocity of the E' wave at the mitral annulus is normal, with a decreased E' wave velocity at the lateral wall, annulus reversus, possibly due to pericardial calcifications or adhesions. Mild mitral regurgitation and mild aortic regurgitation are detected. The right chambers are not dilated, the right ventricle is small, but with altered longitudinal systolic function, as indicated by Tricuspid Annular Plane Systolic Excursion (TAPSE) = 6.5 mm. Mild tricuspid regurgitation with Pulmonary Artery Systolic Pressure (PASP) = 36 mmHg. Possible secondary pulmonary hypertension. Enlarged inferior vena cava without inspiratory collapse (Figure 6).

**Figure 7:**
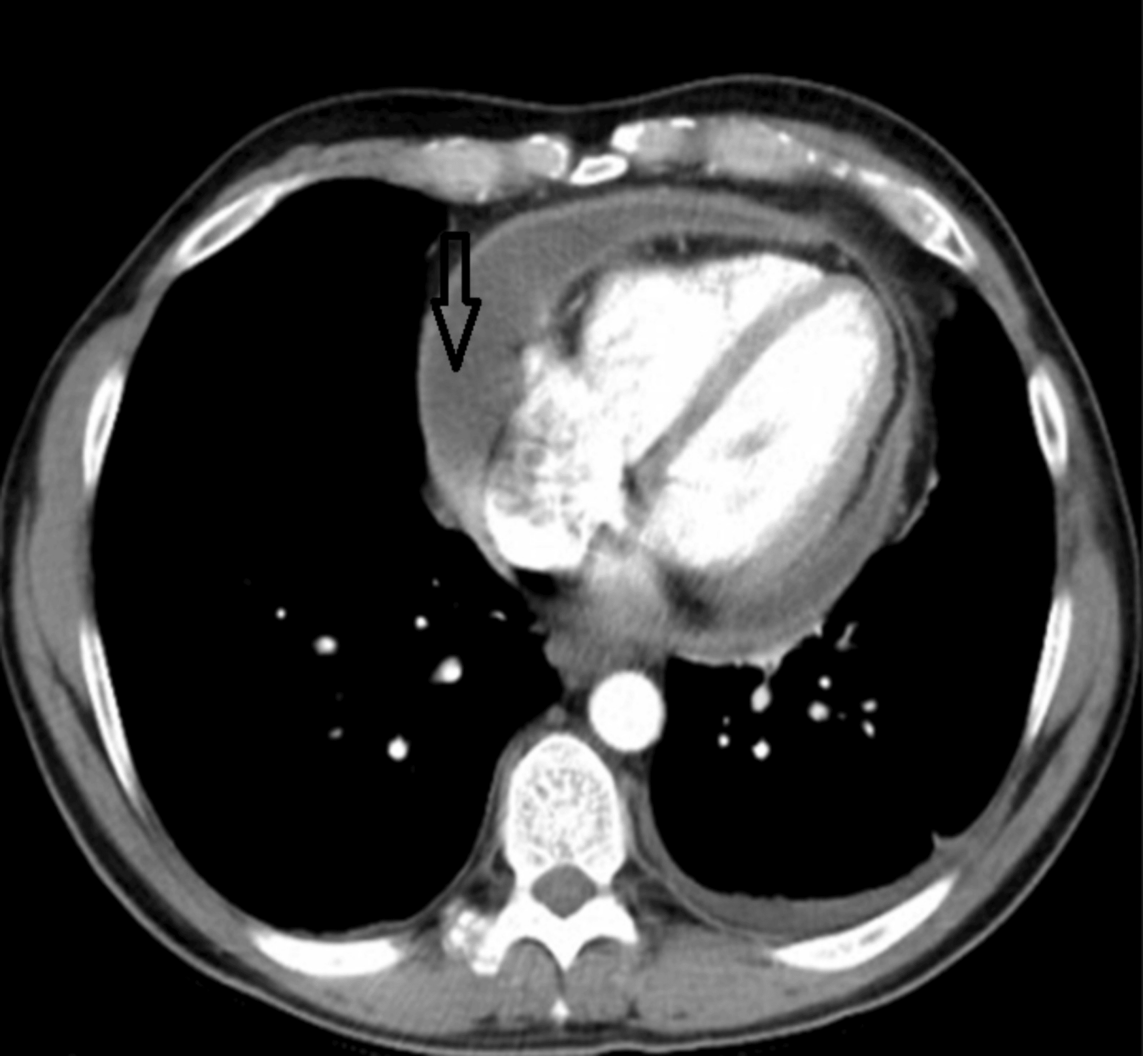
Chest CT scan (axial) The image shows pericardial fluid with a maximum size of 3.04 cm.

The interventricular and interatrial septa were intact, with no attached formations or intracavitary thrombi. Chest and abdominal CT images revealed pulmonary parenchyma without areas of consolidation or suspicious nodules. Apical and posterobasal fibrosis lesions bilaterally. Mediastinal adenopathy with maximum dimensions of 2.4/1.3 cm subaortic. Left pleural fluid with a maximum thickness of 2.4 cm and a thin layer of fluid at the right basal area was observed. Pericardial fluid with a maximum size of 3.04 cm, associated with mild circumferential pericardial thickening, was observed. During the longitudinal pericardiectomy, a markedly thickened and inflamed parietal pericardium was identified, with a maximum thickness of 8 mm. Pericardial nodules were observed, the largest measuring 1.1 cm in diameter, consistent with preoperative imaging findings (Figure 7). Multiple hepatic hemangiomas, minimal pericolecystic fluid.

Coronary Computed Tomography (CT) Angiography was performed with acquisition and post-processing protocols for the elective study of coronary circulation. Calcium score was found to be 0. All of these observations concluded epicardial coronary arteries without significant stenoses, anterior pericardial fluid of the right ventricle with a maximum thickness of 29 mm, exerting a compressive effect on the pericardium.

**Figure 8:**
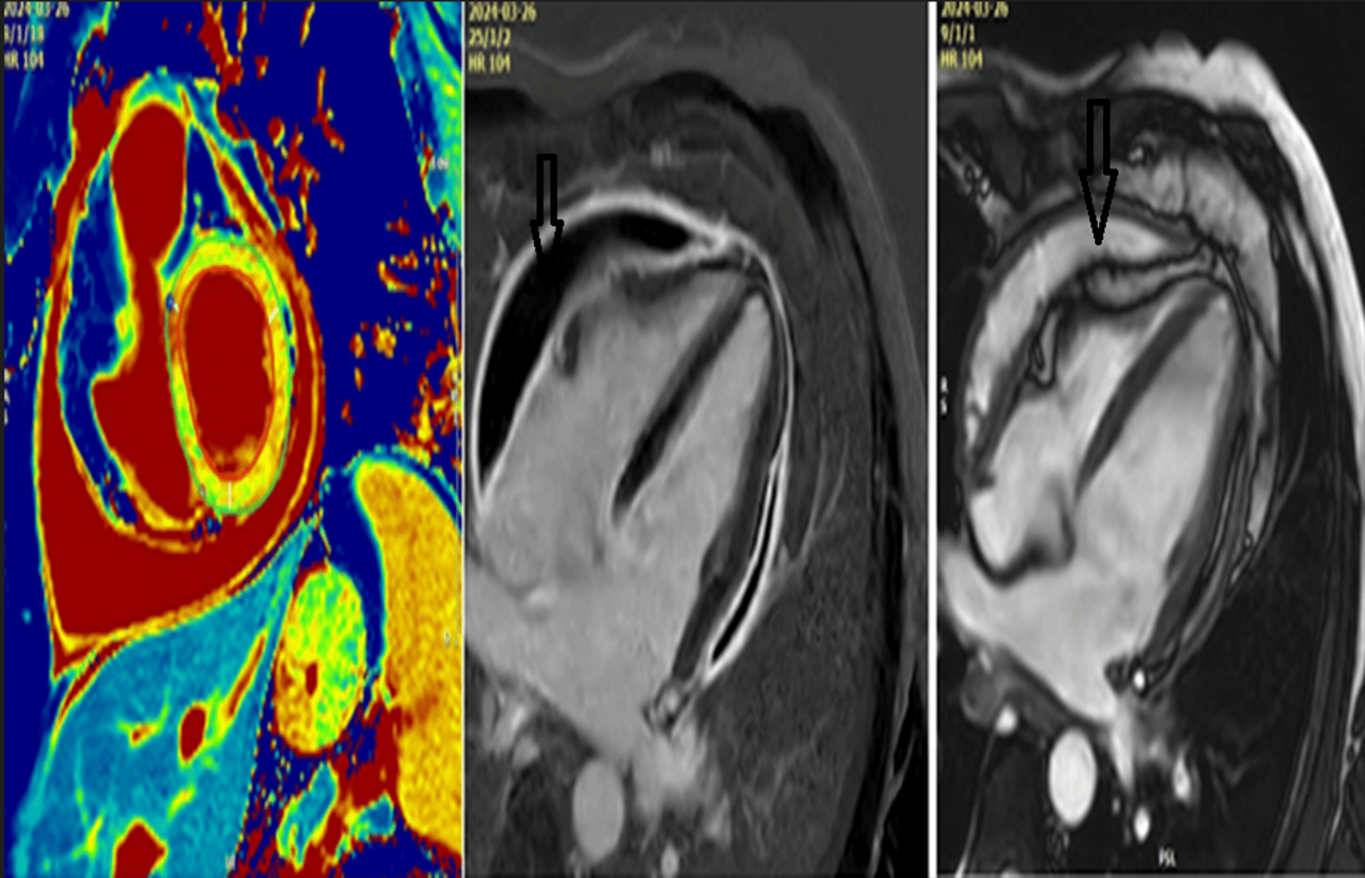
Cardiac MRI Cardiac MRI confirms the diagnosis of effusive constrictive pericarditis

Per cardiac MRI summary, the left ventricle was small in size with moderate dysfunction (left ventricular ejection fraction (LVEF) = 36%). The right ventricle is small in size with moderate dysfunction (right ventricular ejection fraction (RVEF) = 37%). No fibrosis was detected at the myocardial level, but diffuse impairment due to increased T1 was observed. Severely thickened pericardium, causing significant restriction of biventricular relaxation, was seen. Moderate pericardial fluid was distributed in the infero-posterior portion. Pericardial edema and pericardial enhancement were observed. The T1 value under 3000 ms of the pericardial fluid suggests the presence of exudate. Cardiac MRI confirmed the diagnosis of constrictive pericarditis with inflammatory features, moderate effusion, and significant impairment of biventricular function (Figure 8). After the preoperative protocol was completed, approximately 14 days later, it was decided to admit the patient to a cardiovascular surgery center for the resolution of the case.

**Figure 9:**
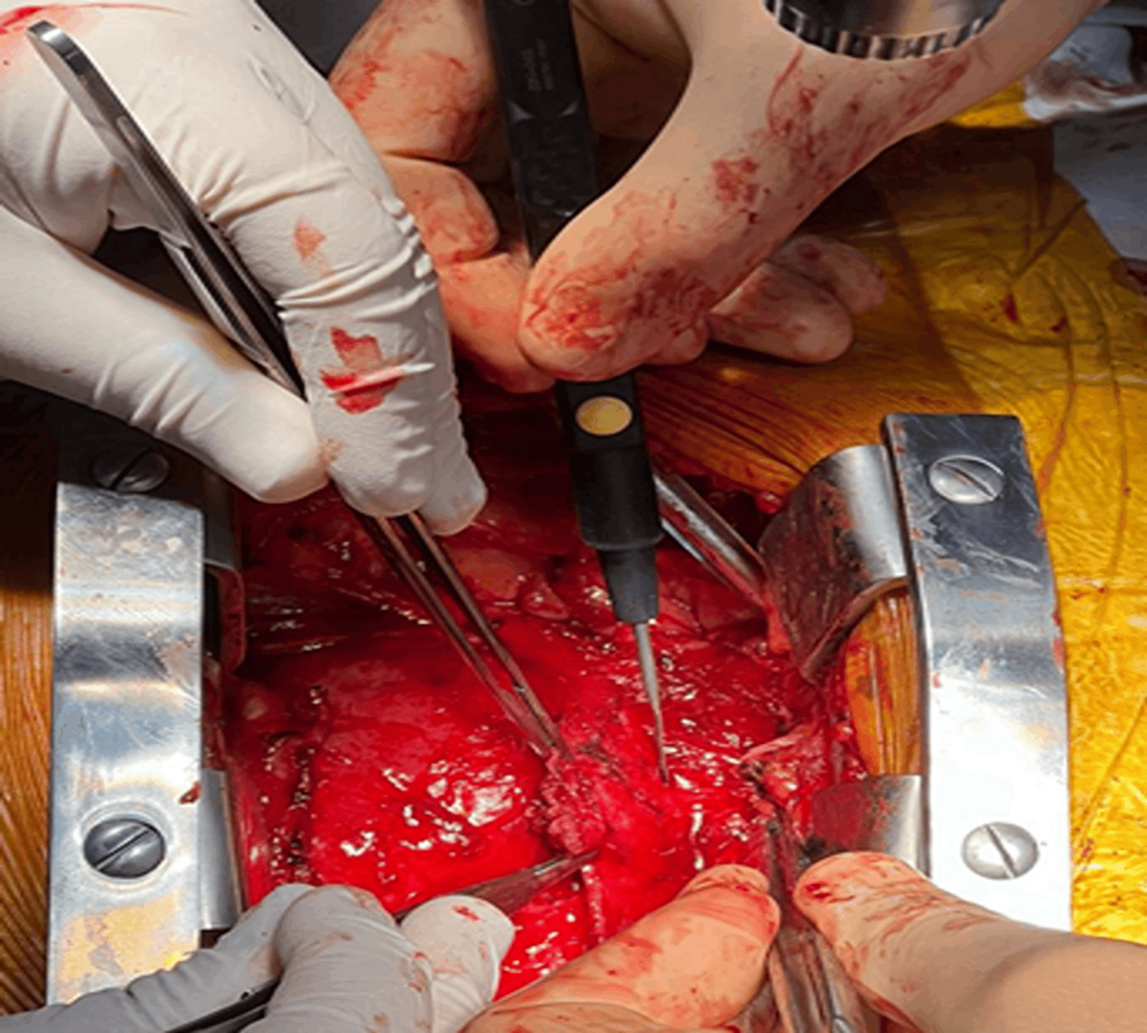
Intraoperative image

Surgical protocol involved extensive anterior and lateral pericardiectomy. During longitudinal pericardiectomy, a thickened, inflamed parietal pericardium is observed. Maximum thickness of 8 mm, pericardial nodules with a maximum size of 1.1 cm, similar to imaging findings. Debridement was performed, and the release of the parietal pericardium from the visceral pericardium began anteriorly, then laterally bilaterally (Figure 9). The right atrium and right ventricle were released, and partially the left ventricle. The parietal pericardium was resected up to the level of the phrenic nerves. Laterally to the right atrium, an abscess with a caseous necrosis appearance was observed. The caseous necrosis observed in the pericardium was characterized by a whitish-yellow area with a friable and soft texture. The affected tissue exhibited a granular consistency that easily crumbled and disintegrated upon manipulation. Specimens were sent for histopathological examination. The pleural cavities were opened, and serocitrin fluid was aspirated. Drain tubes were placed. Sternum closure was done, and the wound was closed in anatomical layers.

After the surgical intervention, the patient's symptoms were eased, with a decrease in inflammatory markers and improvement in the imaging appearance upon echocardiographic visualization. Histopathological examination revealed dense fibrous thickening of the pericardial layers with areas of hyalinization and minimal chronic inflammatory infiltrate. No granulomas or specific infectious agents were identified. The histopathological specimens did not identify a specific cause, suggesting an idiopathic constrictive pericarditis. 

During periodic postoperative evaluations, at three and six months, the patient shows progressive improvement of symptoms, with the disappearance of fatigue and reduction of dyspnea. Clinical examination reveals hemodynamic stability without signs of heart failure. Echocardiography confirms remission of pericardial constriction and normal ventricular function. Functional capacity improves, allowing the patient to resume daily activities without limitations. Ongoing monitoring has not revealed any recurrences or complications to date.

## Discussion

As a disease of the pericardium, its main characteristic is the presence of chronic inflammation or scarring, or their combination, as well as the adherence of the pericardium to the myocardium and the diastolic expansion of both ventricular walls [3]. In constrictive pericarditis, the pericardium loses its elasticity due to chronic inflammatory processes, leading to thickening, extensive fibrosis, and sometimes calcifications. These changes transform the pericardium into a rigid layer that prevents normal filling during diastole [4]. Blood flow to the heart is limited, diastolic pressures rise rapidly, and cardiac function depends on a fixed volume [4]. The essential pathophysiological phenomenon is ventricular interdependence, associated with the appearance of a specific pressure pattern known as the dip and plateau [4]. Constrictive pericarditis was historically considered a surgical disease, but research indicates that some forms, such as the transient type, can be treated medically [3]. An article from 2011 explores the risk of constrictive pericarditis as a rare but serious complication of acute pericarditis [5].

The authors emphasize that constrictive pericarditis is more commonly associated with certain etiologies, such as tuberculosis or purulent pericarditis, but it is less likely to occur in cases of viral or idiopathic pericarditis. In a long-term prospective study, 500 patients with acute pericarditis were analyzed [5]. The results show that only 1.8% of patients developed constrictive pericarditis, and the risk varies significantly depending on the cause of pericarditis; less than 0.5% for viral or idiopathic etiologies, but much higher for bacterial infections or neoplastic diseases [5]. This highlights the importance of accurately diagnosing the underlying cause and long-term monitoring to detect the progression to constrictive pericarditis at an early stage [5]. Constrictive pericarditis can have multiple etiologies, the most common being viral or idiopathic pericarditis, tuberculosis, especially in regions with high prevalence, thoracic radiotherapy, previous cardiac surgeries, and connective tissue diseases such as systemic lupus erythematosus. Other causes include bacterial or fungal infections, uremia, and malignant conditions [4]. Tuberculosis remains one of the main causes worldwide; however, in developed countries, non-infectious etiologies, such as radiotherapy or previous cardiac surgeries, are on the rise. Approximately 40% of cases are idiopathic, meaning no clear cause is identified, and pericardial fibrosis may result from chronic inflammation or autoimmune processes [4]. An infrequent cause of constrictive pericarditis is genetic, as described in a rare case of familial constrictive pericarditis associated with the CACP syndrome (camptodactyly-arthropathy-coxavara-pericarditis) [6]. The study details the clinical history of two siblings with multiple joint deformities and constrictive pericarditis, genetically confirmed to be caused by the presence of a rare mutation in the PRG4 gene, responsible for the production of lubricin, an essential lubricant for the joints and pericardium. The study emphasizes the importance of genetic testing in identifying rare mutations that contribute to complex hereditary diseases [6]. There have been cases of constrictive pericarditis described following SARS-CoV-2 vaccination, especially in adolescents or young adults [7]. Patients with effusive-constrictive pericarditis present clinical symptoms that combine the characteristics of pericardial tamponade with signs of constriction. These include dyspnea, fatigue, and signs of right heart failure such as jugular distention, peripheral edema, and hepatomegaly [8].

In two-dimensional echocardiography, an essential investigation in the diagnosis of constrictive pericarditis, the thickened pericardium can be visualized, and the abnormal movement of the interventricular septum, referred to as bouncing septum, is another suggestive sign, present in up to 96% of patients with constrictive pericarditis, best visualized in M-mode [4,9]. Tissue Doppler Imaging of the E' wave at the medial and lateral mitral annulus is of particular importance in constrictive pericarditis. Thus, a larger E' wave at the medial level compared to the lateral level is strongly associated with constrictive pericarditis [10]. This is possible due to the presence of calcifications in the pericardium or its adhesions. In cases of constrictive pericarditis, the E' velocity is often retained or increased, and this preservation of E' has been identified as a helpful clinical indicator to distinguish constrictive pericarditis from restrictive cardiomyopathy [11]. The hepatic veins provide very important details through the dissociation of intrathoracic and intracardiac pressures and the ventricular interdependence through the reversal of the expiratory diastolic flow in patients with constrictive pericarditis [10]. Additionally, the presence of a dilated inferior vena cava, which does not collapse during inspiration, is an indicator of constrictive pericarditis [4-12]. Another significant aspect of pulsed Doppler echocardiography is the modification of the transmitral and transtricuspid flow based on respiration. In constrictive pericarditis, the transmitral flow decreases during inspiration and increases during expiration, while the opposite phenomenon occurs in the transtricuspid flow [4]. The modification of the flow can be an important sign of constrictive pericarditis when the decrease in the amplitude of the E wave of the transmitral flow exceeds 25%. Thus, echocardiography provides valuable information and significantly contributes to the diagnosis of constrictive pericarditis, facilitating the identification of the characteristic signs of this condition [4].

The American College of Cardiology recommends performing cardiac MRI in patients with pericardial involvement who have symptoms lasting more than three months and elevated inflammatory markers [9]. Specific sequences performed through cardiac magnetic resonance help in completing the diagnosis of constrictive pericarditis [13]. Using multiple diagnostic methods aids in distinguishing constrictive pericarditis from other diseases. Cardiac magnetic resonance imaging is crucial in this process, while invasive hemodynamic assessments are necessary to establish a definitive diagnosis. A pericardial thickness greater than 4 mm is considered pathological. The thickness of the pericardium and the modification of the transmitral flow during inspiration represent 90% specificity and 100% sensitivity for constrictive pericarditis [13]. Additionally, in evaluating hemodynamic changes and detecting pericardial thickening, the cardiac MRI technique visualizes the increased displacement of the interventricular septum in response to respiratory variations. It also highlights the presence of pericardial edema and inflammation [14]. The presence of these changes in the pericardium suggests an active process that is suitable for anti-inflammatory therapy [14]. Computed tomography, a complementary diagnostic tool with a prognostic role, is sensitive in detecting pericardial thickening or calcifications. Although cardiac CT is not typically the primary diagnostic tool for suspected constrictive pericarditis, it proves valuable in surgical planning for pericardiectomy. Multidetector CT provides important details such as the precise positioning of cardiac and vascular structures in relation to the midline behind the sternum, as well as detecting aortic atherosclerotic changes. Not all patients with pericardial calcifications have constrictive pericarditis, and approximately one-third of patients with constrictive pericarditis do not have thickened pericardium [14]. Cardiac catheterization can confirm the diagnosis and can be performed preoperatively [15]. For elderly patients with moderate constrictive pericarditis, conservative therapy can be managed through the administration of diuretics. The definitive treatment for constrictive pericarditis remains pericardiectomy [15]. Due to the danger associated with chronicization and unfavorable progression, pericardiectomy is recommended early in patients with constrictive pericarditis. Patients who undergo pericardiectomy have a favorable evolution with improved survival [15]. Median sternotomy is preferred because it allows for a more thorough cleaning of the pericardium. Approximately 40% of patients undergoing pericardiectomy for constrictive pericarditis experience a return to normal left ventricular diastolic function, and 60% of patients have normal diastolic function over time. Unfavorable post-pericardiectomy outcomes have been associated with incomplete resection of the pericardium, myocardial fibrosis, long-standing pericardial disease, and cardiac compression [15]. Regarding the biopsy results in patients with constrictive pericarditis, according to a 2003 study, the results were nonspecific, and no restrictive myocardial diseases were identified [16].

The prognostic factors of constrictive pericarditis were analyzed based on a study that included 140 patients who underwent pericardiectomy. The main risk factors for a poorer prognosis are advanced age at the time of surgery, advanced functional class NYHA III-IV, and a history of idiopathic acute pericarditis [17]. In the Cox regression analysis, these factors were correlated with an increased mortality rate during the postoperative follow-up period, which averaged 12 years. No significant differences in long-term mortality were observed based on the etiology of the pericarditis [17].

## Conclusions

Constrictive pericarditis remains a challenging diagnosis due to its often nonspecific clinical presentation and variable etiology. This case underscores the importance of maintaining a high index of suspicion, especially in younger patients presenting with signs of heart failure and elevated inflammatory markers without an obvious underlying cause. Multimodal imaging, especially echocardiography, cardiac MRI, and CT, plays a pivotal role in establishing a definitive diagnosis and in ruling out differential causes, such as ischemic heart disease or malignancy. In this case, the integration of clinical, laboratory, and advanced imaging data enabled a timely and accurate diagnosis of idiopathic constrictive pericarditis.

While pericardiectomy is considered the definitive and potentially curative treatment in patients with preserved myocardial function and a favorable long-term prognosis, outcomes are generally less predictable in those with impaired cardiac function. In our case, early referral of a young patient with biventricular dysfunction and timely surgical intervention in a specialized center were crucial in achieving a positive outcome. Ultimately, this case highlights the need for vigilance in identifying constrictive pericarditis and emphasizes the role of coordinated, multidisciplinary management in improving both symptoms and long-term prognosis, especially in young, otherwise healthy individuals.
